# Supplementary material for: An optogenetic approach for regulating human parathyroid hormone secretion
Source: Nat Commun. 2022 Feb 9;13:771. doi: 10.1038/s41467-022-28472-9 (PMC8828854; doi:10.1038/s41467-022-28472-9)
Supplement: Supplementary file 1 — Supplementary information [file 41467_2022_28472_MOESM1_ESM.pdf]

Supplementary Figure. 1

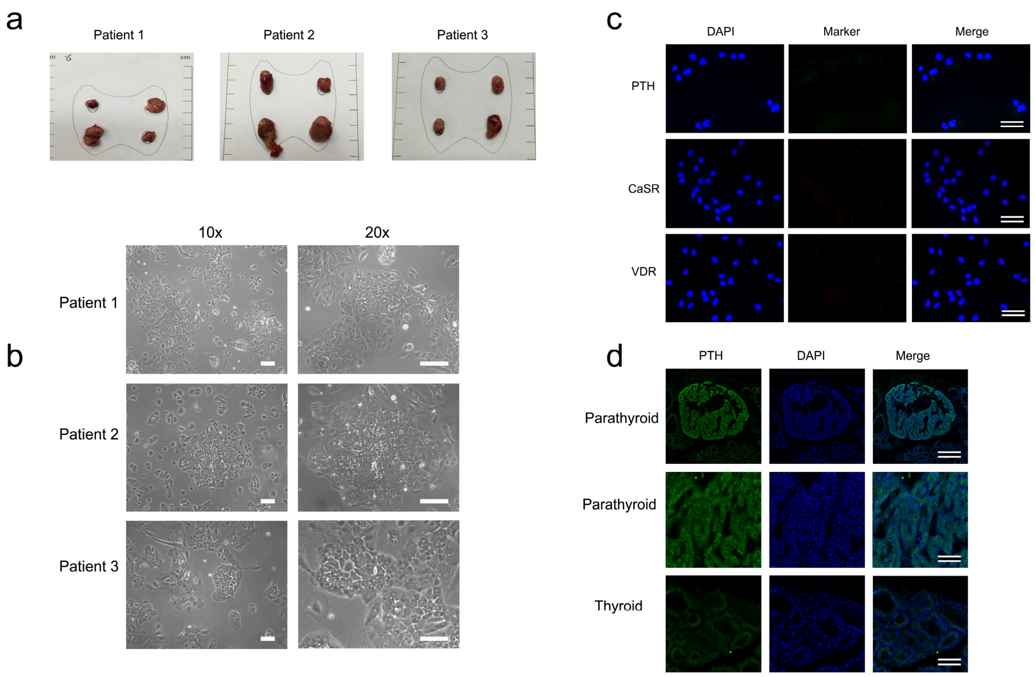

Supplementary Fig. 1 Isolation and culture of parathyroid cells from patients with secondary hyperparathyroidism (SHPT)

a, Parathyroid glands were isolated from three representative patients with a diagnosis of SHPT. **b,** Cultured parathyroid cells from three patients reached 70% confluence after seven days of cultivation and the cell shape appeared uniform and maintained epithelial cell-like morphology under different magnifications. Scale bar=100 μm. **c,** Negative control of immunostaining of parathyroid hormone (PTH), calcium sensing receptor (CaSR) and Vitamin D receptor (VDR) on representative cultured human parathyroid cells. Scale bar=50 μm. **d,** Immunostaining of parathyroid hormone (PTH) on rat parathyroid gland and thyroid gland tissue. Scale bar=100 μm.

Supplementary Figure. 2

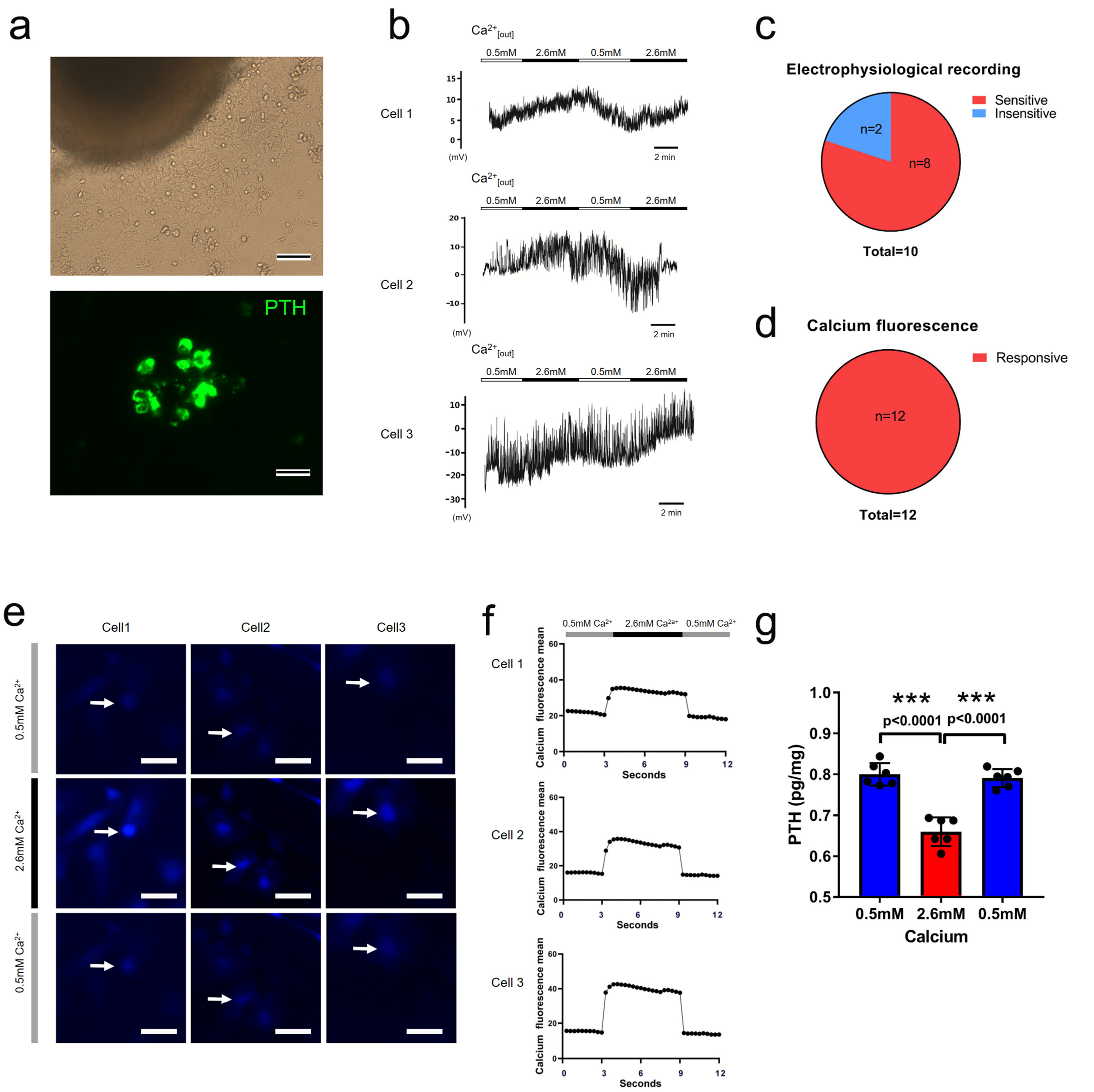

Supplementary Fig. 2 Normal rat parathyroid cells respond to changes in extracellular calcium

a, Parathyroid glands were isolated from normal rat, and cultured parathyroid cells reached 70% confluence after seven days of cultivation. Immunostaining of parathyroid hormone (PTH) in representative cultured rat parathyroid cells. Scale bar=100 μm (top); 40 μm (bottom). **b,** Electrophysiological recordings of the membrane potential of three representative parathyroid chief cells during the change of extracellular Ca^{2+} from 0.5 to 2.6 mM. **c,** Summary of electrophysiological recording statistics from 10 parathyroid

cells from 3 rats; 8 of 10 cells were sensitive to high extracellular calcium challenge. **d,** In normal rat parathyroid, 12 of 12 cells from 3 rats were sensitive to extracellular Ca^{2+} and showed fluctuations of fluorescence during extracellular Ca^{2+} changes. **e,** Calcium fluorescence assay reflecting the change of the intracellular Ca^{2+} concentrations in three representative parathyroid cells responding to extracellular Ca^{2+}. Calcium indicator dye Fura-2-AM was used to reveal intracellular Ca^{2+} changes at 0.5 and 2.6 mM extracellular Ca^{2+}. Scale bar=20 μm. **f,** Quantification of the calcium fluorescence intensity over time in 3 representative parathyroid chief cells. **g,** Quantification of the level of PTH secreted from cultured rat parathyroid cells at 0.5 mM extracellular Ca^{2+} and 2.6 mM extracellular Ca^{2+} (n=6 per group). Two-tailed unpaired *t* test, $p<0.0001$. Values represent mean ± SEM. Source data are provided as a Source Data file.

Supplementary Figure. 3

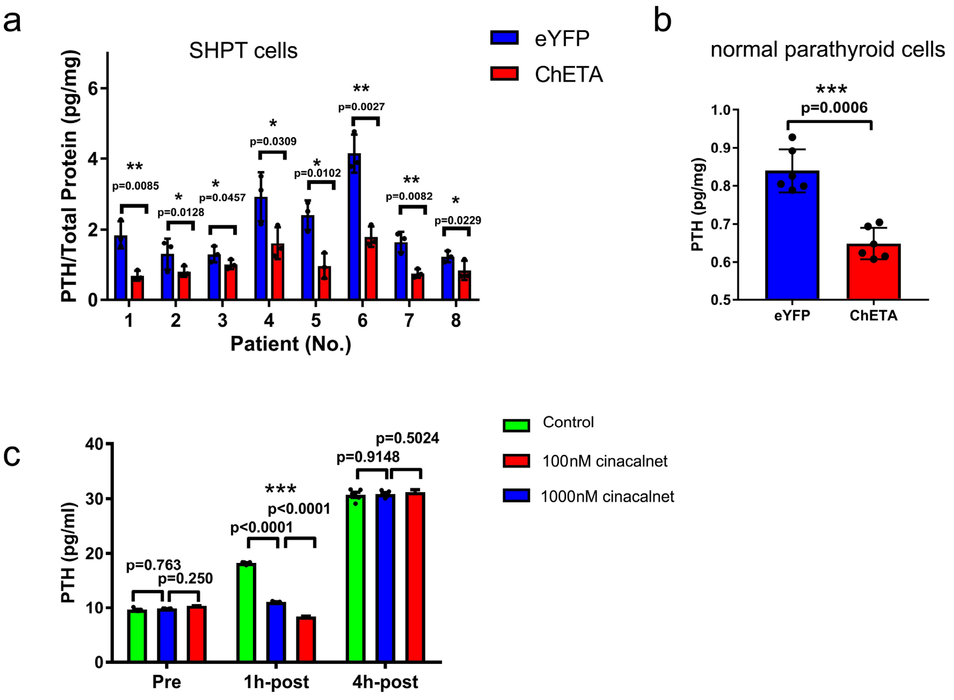

Supplementary Fig. 3 Blue-light stimulation and cinacalcet inhibits secretion of PTH in parathyroid cells

a, Quantification of PTH levels was performed on the cultured medium from stimulated human parathyroid cells of eight patients with SHPT. PTH levels were unanimously lower in the ChETA group than in the control eYFP group at 1h after light stimulation (n=3 per group). **b,** Quantification of the level of PTH secreted from cultured normal rat parathyroid cells after the optogenetic regulation (n=6 per group). **c,** Quantification of PTH level in the cultured medium from cinacalcet treated human parathyroid cells (n=5 per group). All statistical tests in **a-c** used: two-tailed unpaired *t* test, *p* values as indicated. Values represent mean ± SEM. Source data are provided as a Source Data file.

Supplementary Figure. 4

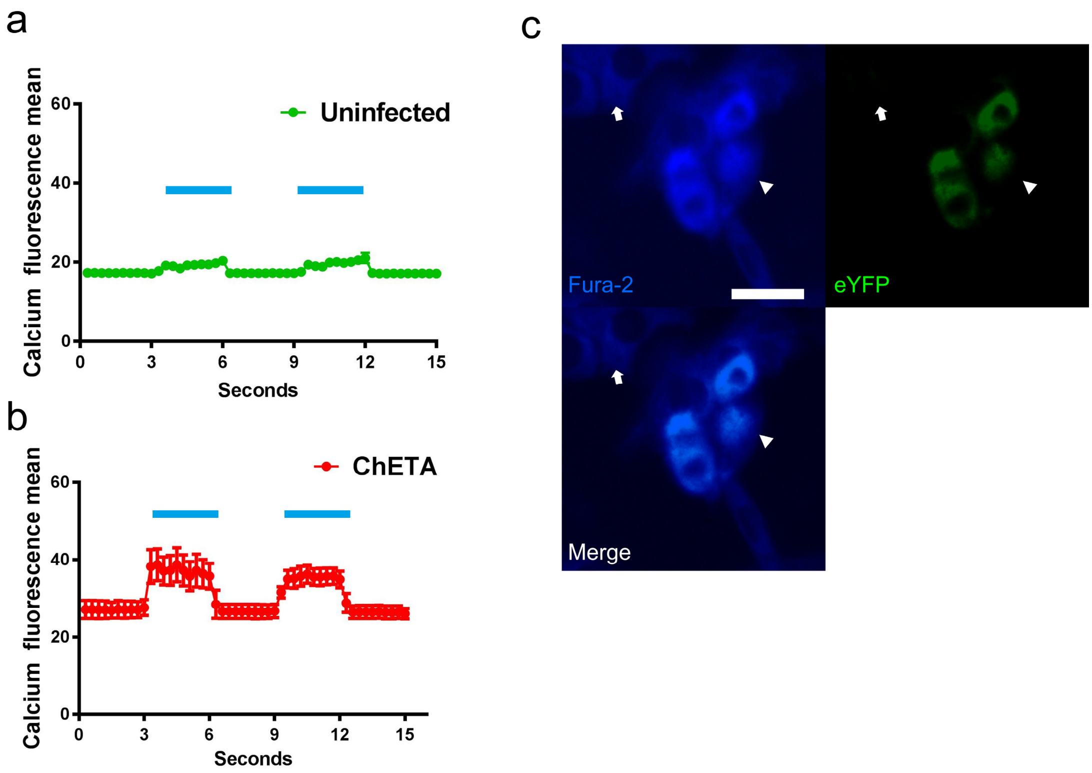

Supplementary Fig. 4 a, **b**, Quantification of calcium fluorescence signals with time in uninfected and ChETA-expressing cells. Blue lines indicate the timepoint of blue light stimulation. Values represent mean ± SEM. **c**. Arrows show an uninfected cell; arrow heads show a ChETA-expressing cell. Scale bar= 20um. Source data are provided as a Source Data file.

Supplementary Figure. 5

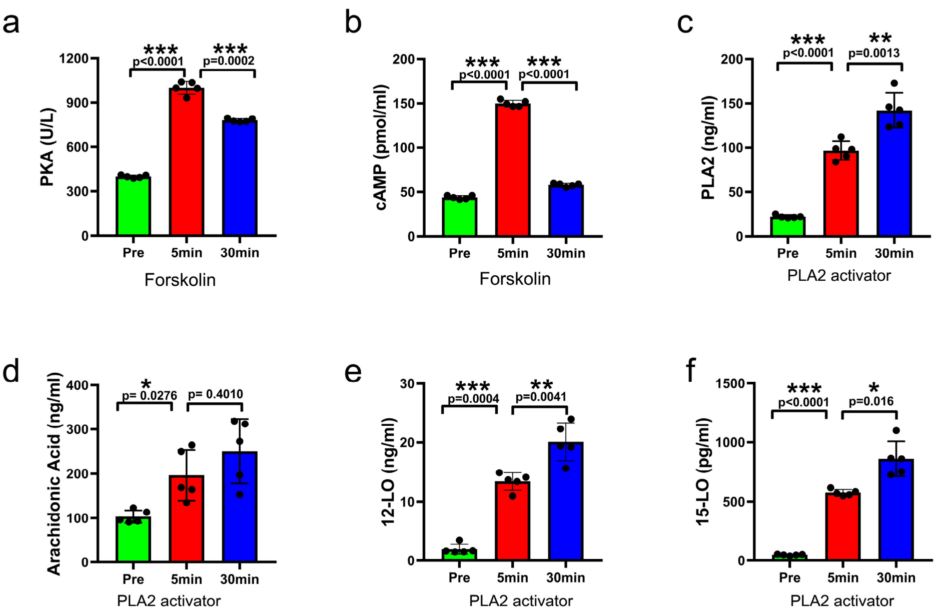

Supplementary Fig. 5 The molecular pathways regulating PTH synthesis and secretion

a, Quantification of PKA level before (Pre), 5 min and 30 min after forskolin (n=5 per group). **b,** Quantification of cAMP levels before (Pre), 5 min and 30 min after forskolin (n=5 per group). **c,** Quantification of Phospholipases A2 (PLA2) in the before (Pre), 5 min and 30 min after PLA2 activator (n=5 per group). **d,** Quantification of arachidonic acid (AA) before (Pre), 5 min and 30 min after PLA2 activator (n=5 per group). **e,** Quantification of 12-lipoxygenase (12-LO) before (Pre), 5 min and 30 min after PLA2 activator (n=5 per group). **f,** Quantification of 15-lipoxygenase (15-LO) before (Pre), 5 min and 30 min after PLA2 activator (n=5 per group). All statistical tests in **a-f** used: two-tailed unpaired *t* test, *p* values as indicated. Values represent mean ± SEM. Source data are provided as a Source Data file.

Supplementary Figure. 6

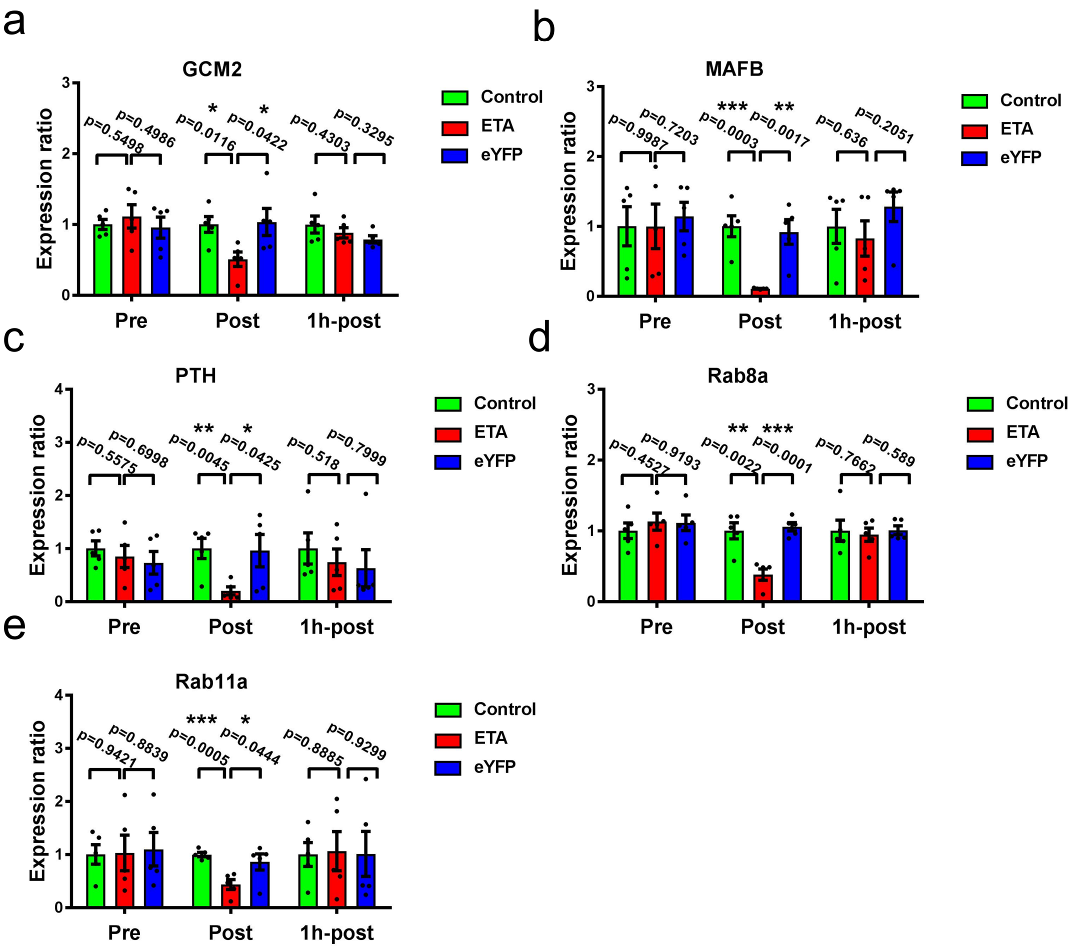

Supplementary Fig. 6 RT-PCR analysis of key elements of PTH production and vesicle trafficking in PTG cells before and after light stimulation.

a, *GCM2* levels in the control, eYFP and ChETA groups before (Pre), immediate after (Post) and 1 h after light stimulation (1h-post) values represent mean ±SEM (n=5 per group); **b**, *MAFB* in the control, eYFP and ChETA groups before (Pre), immediate after (Post) and 1 h after light stimulation (1h-post); values represent mean ±SEM (n=5 per group); **c**, *PTH* in the control, eYFP and ChETA groups before (Pre), immediate after (Post) and 1 h after the light stimulation (1h-post); values represent mean ±SEM (n=5 per group); **d**, *Rab8a* in the control, eYFP and ChETA groups before (Pre), immediate

after (Post) and 1 h after the light stimulation (1h-post). Values represent mean ±SEM (n=5 per group); **e**, *Rab11a* in the control, eYFP and ChETA groups before (Pre), immediate after (Post) and 1 h after the light stimulation (1h-post); values represent mean ±SEM (n=5 per group). All statistical tests in **a-e** used: one-way analysis of variance (ANOVA) with Tukey's multiple comparisons test, *p* values as indicated. Values represent mean ± SEM. Source data are provided as a Source Data file.

Supplementary Figure. 7

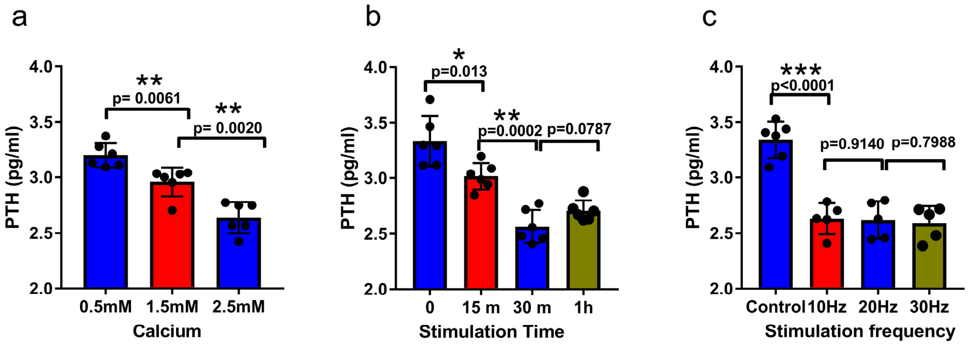

Supplementary Fig. 7 Extracellular calcium and optogenetic stimulation of normal rat parathyroid cells inhibit the secretion of PTH

a. Quantification of PTH levels secreted from cultured rat parathyroid cells at 0.5 mM, 1.5 mM and 2.5 mM extracellular Ca^{2+} (n=6 per group). **b.** Quantification of PTH levels secreted from normal rat parathyroid cells at 0 (before), 15 min, 30 min and 1 h after stimulation with 20-Hz blue light (n=6 per group). **c.** Quantification of PTH levels 30 min after stimulation with 10- and 20-Hz blue light (n=5 per group); All statistical tests in **a-c** used: two-tailed unpaired *t* test, *p* values as indicated. Values represent mean ± SEM. Source data are provided as a Source Data file.

Supplementary Figure. 8

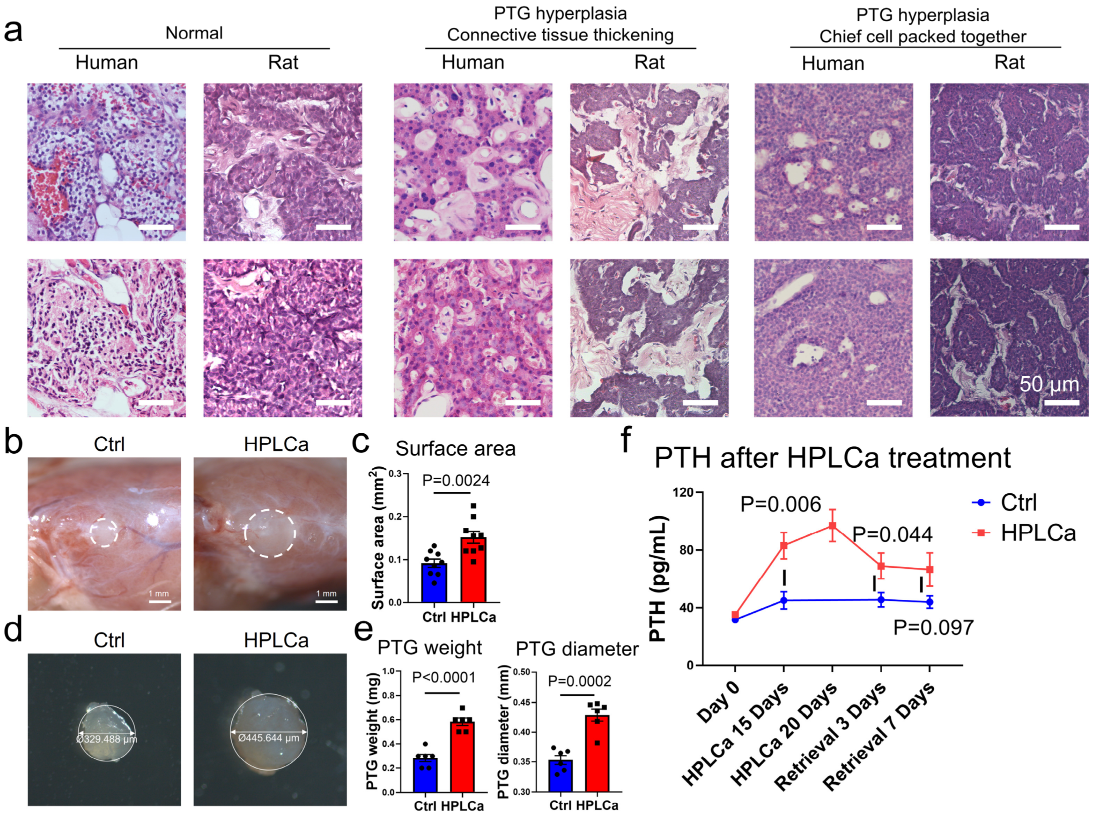

Supplementary Fig. 8 High phosphate and low calcium (HPLCa) diet induces secondary parathyroid hyperplasia in rats. **a**, H&E staining comparing the parathyroid glands of patients and rats. Scale bar=50 μm. **b**, Image of rats' parathyroid glands (PTG) *in situ*. Scale bar=1 mm. **c**, Quantification of rats PTG surface area from *in situ* PTG image (n=9 rats). Two-tailed unpaired *t* test, *p*=0.0024. Values represent mean ± SEM. **d**, Image of isolated rats PTG. **e**, Weight (*left*) and diameter (*right*) of isolated rat PTG. (n=6 rats). **f**, Serum PTH level of rats during the consumption and after the retrieval of HPLCa diet. (n=6 rats). All statistical tests in **e-f** used: Two-tailed unpaired *t* test, *p* values as indicated. Values represent mean ± SEM. Source data are provided as a Source Data file.

Supplementary Figure. 9

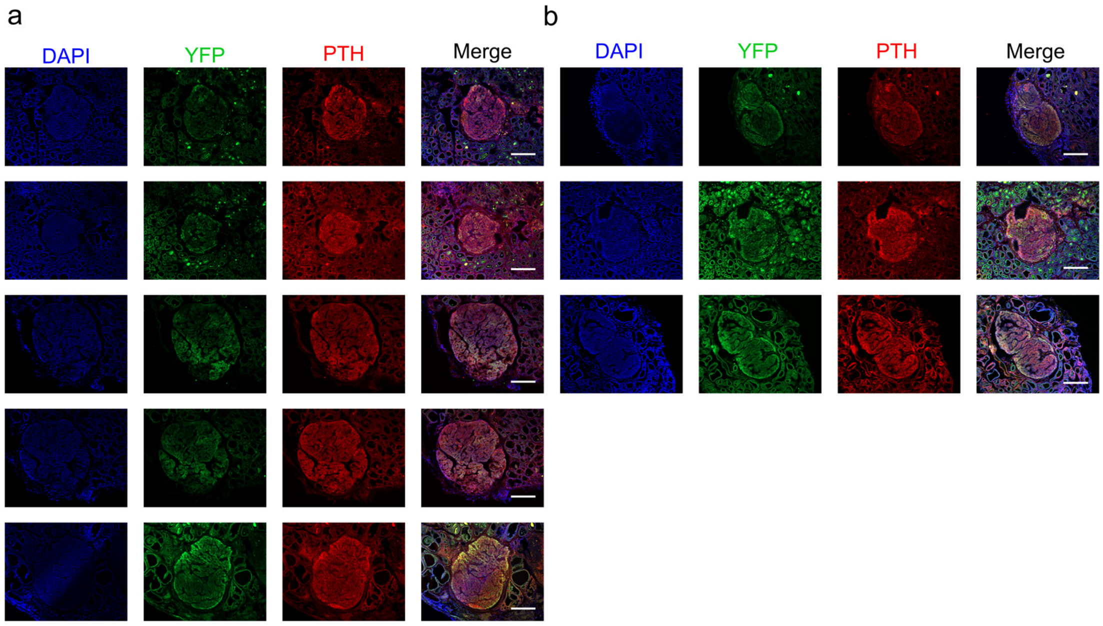

Supplementary Fig. 9 Representative images indicating AAV vector infection rate of rat PTG glands. Immunofluorescent staining of PTH of AAV vector transfected rat PTH of eYFP (**a**, n=5) and ChETA groups (**b**, n=3). Scale bar=200 μm.

Supplementary Figure. 10

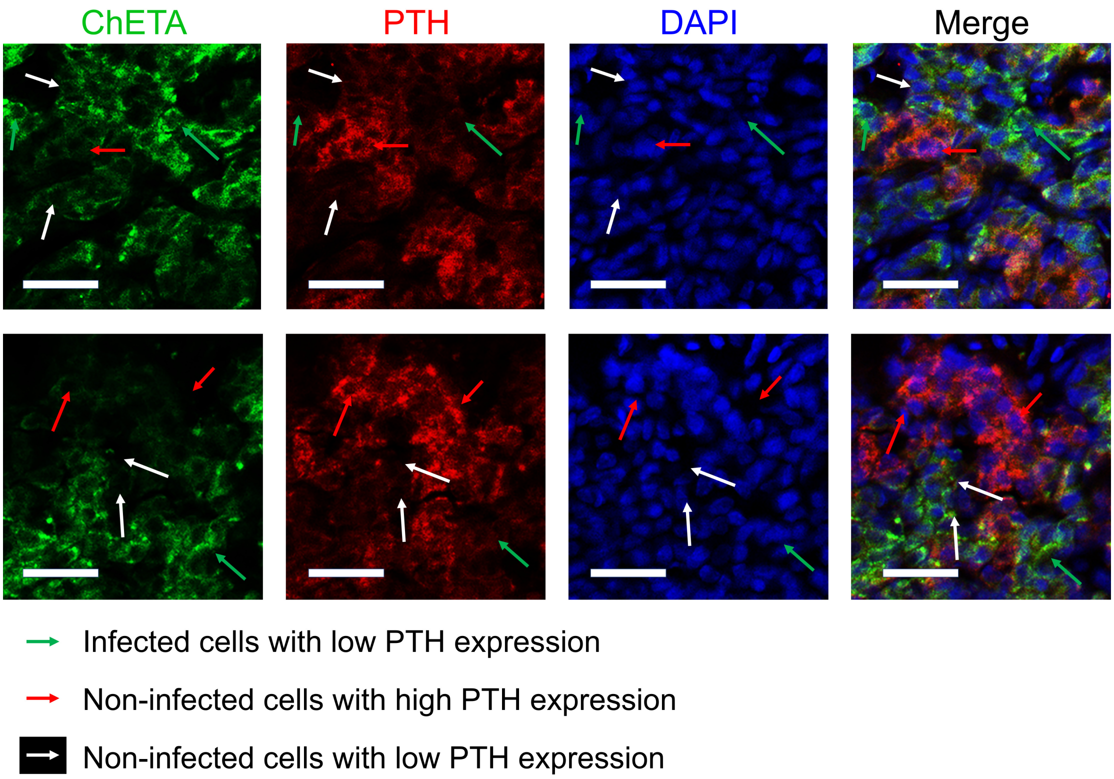

Supplementary Fig. 10 Representative images indicating non-cell autonomous effects were observed in ChETA transfected parathyroid cells. Immunofluorescent staining of PTH of PTG in ChETA groups. The cells with high expression of ChETA had low expression of PTH after light stimulation (green arrow); chief cells with high PTH expression were typically not transfected with ChETA (red arrow); there were also cells that expressed low PTH without expression of ChETA (white arrow). Scale bar=25 μm.

Supplementary Figure. 11

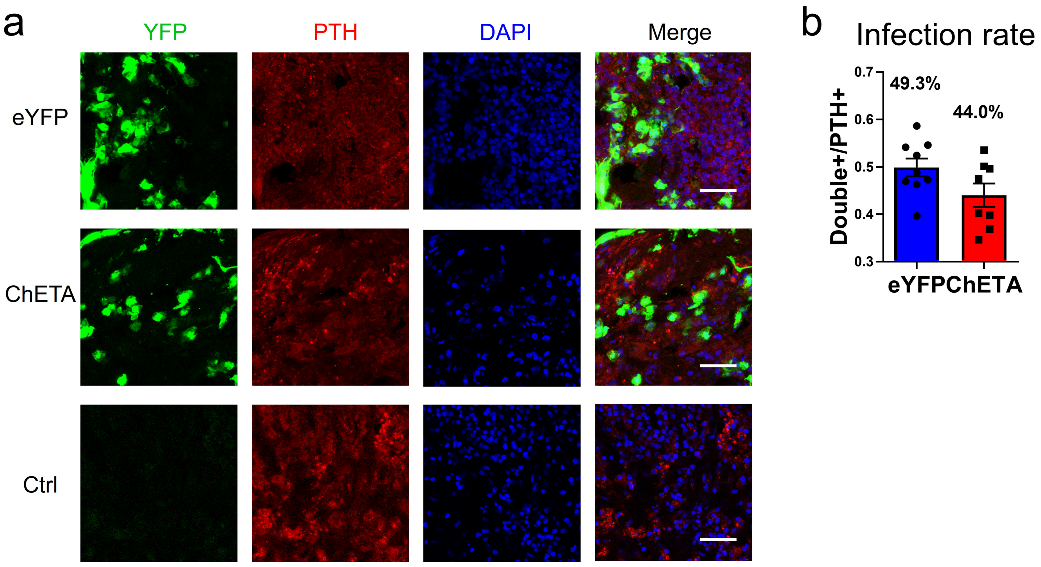

Supplementary Fig. 11 Viral infection rate of human PTG organoids. a, immunofluorescent staining of PTH of AAV vector transfected human PTH graft of control and eYFP and ChETA groups. Scale bar=25 μm. **b**, Infection rate of human PTG graft (n=3 organoids). Values represent mean ± SEM. Source data are provided as a Source Data file.

Supplementary Figure. 12

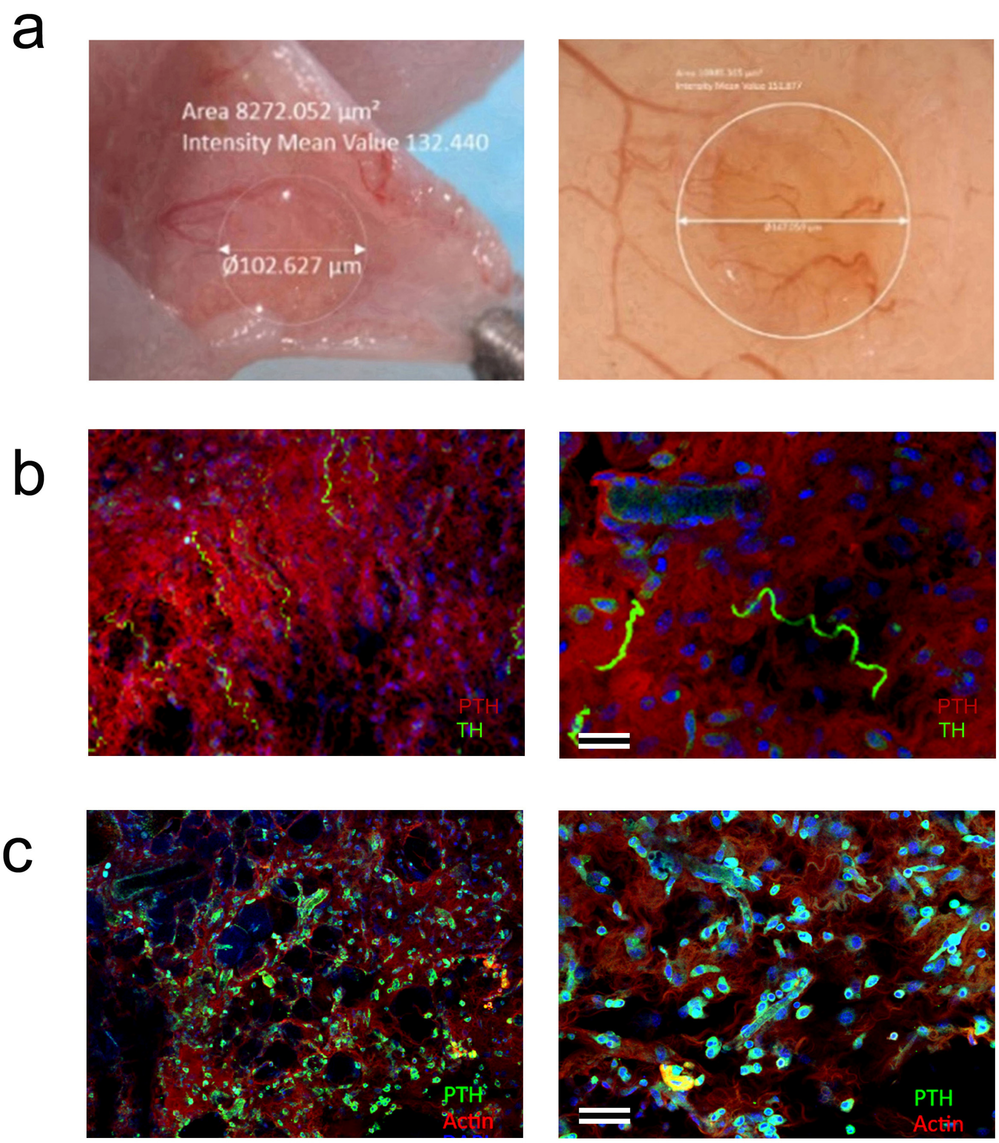

Supplementary Fig. 12 Transplantation and integration of the human parathyroid tissue into nude mice

a, Seven days after transplantation, the size of the parathyroid tissue remained stable and the diameter was around 1 mm. **b,** Immunofluorescence of the tyrosine hydroxylase (TH) and parathyroid hormone (PTH) in the transplanted human parathyroid tissue. Scale bar=50 µm. **c,** Immunofluorescence of alpha-smooth muscle actin and PTH in transplanted human parathyroid tissue. Scale bar=50 µm.

Supplementary Figure. 13

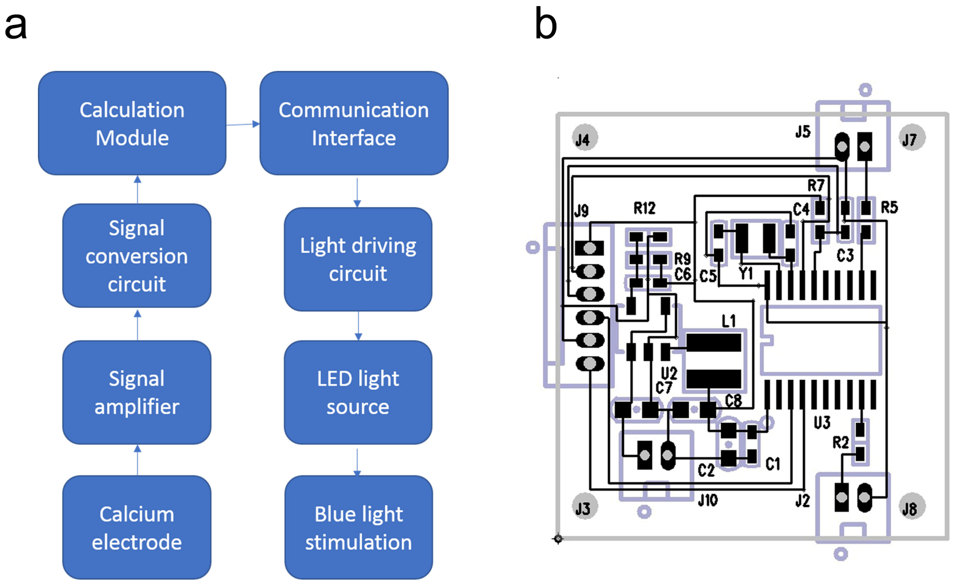

Supplementary Fig. 13 Schematic diagram of calcium monitoring using electrode and blue light stimulation

a, Schematic diagram showing the modules of calcium monitoring using electrode and blue light stimulation. **b,** Schematic electrical circuit of the blue light stimulation device

Supplementary Figure. 14

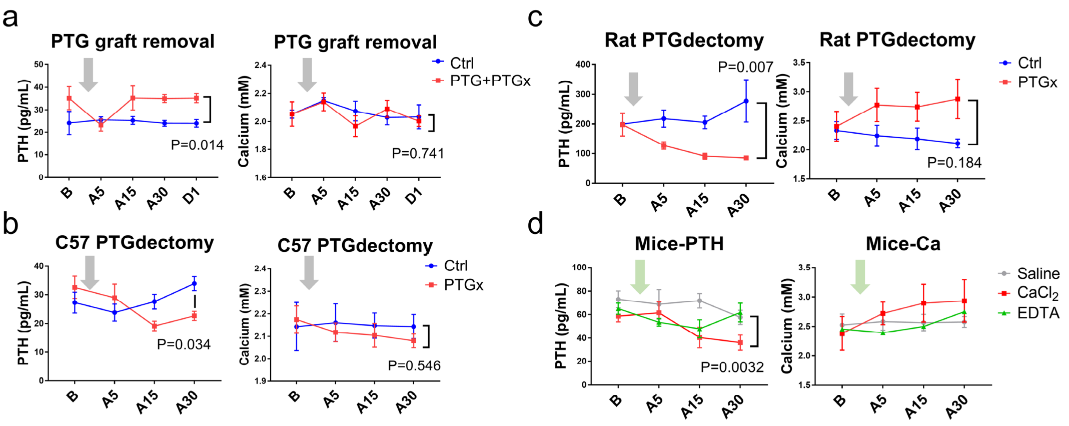

Supplementary Fig. 14 Quantification of serum PTH and calcium after graft removal or parathyroidectomy in different animal models. a, Serum PTH (left) and calcium (right) level of CD-1 nude mice before and after removal of transplanted human PTG graft (n=5 mice per group. left, *p*=0.014; right, *p*=0.0.741). **b**, Serum PTH (left) and calcium (right) level of C57 mice before and after unilateral removal of PTG. (n=5 mice per group. left, *p*=0.034; right, *p*=0.546,). **c**, Serum PTH (*left*) and calcium (*right*) level of sHPT rats before and after unilateral removal of PTG (n=4-5 rats per group. left, *p*=0.007; right, *p*=0.184). **d**, Serum PTH (left) and calcium (right) level of C57 mice before and after *i.v.* injection of saline, $CaCl_2$ and EDTA. (n=4-5 mice per group. left, *p*=0.0032). All statistical tests in **a-d** used: Two-way ANOVA test, *p* values as indicated. Values represent mean ± SEM. Source data are provided as a Source Data file.

Supplementary Figure. 15

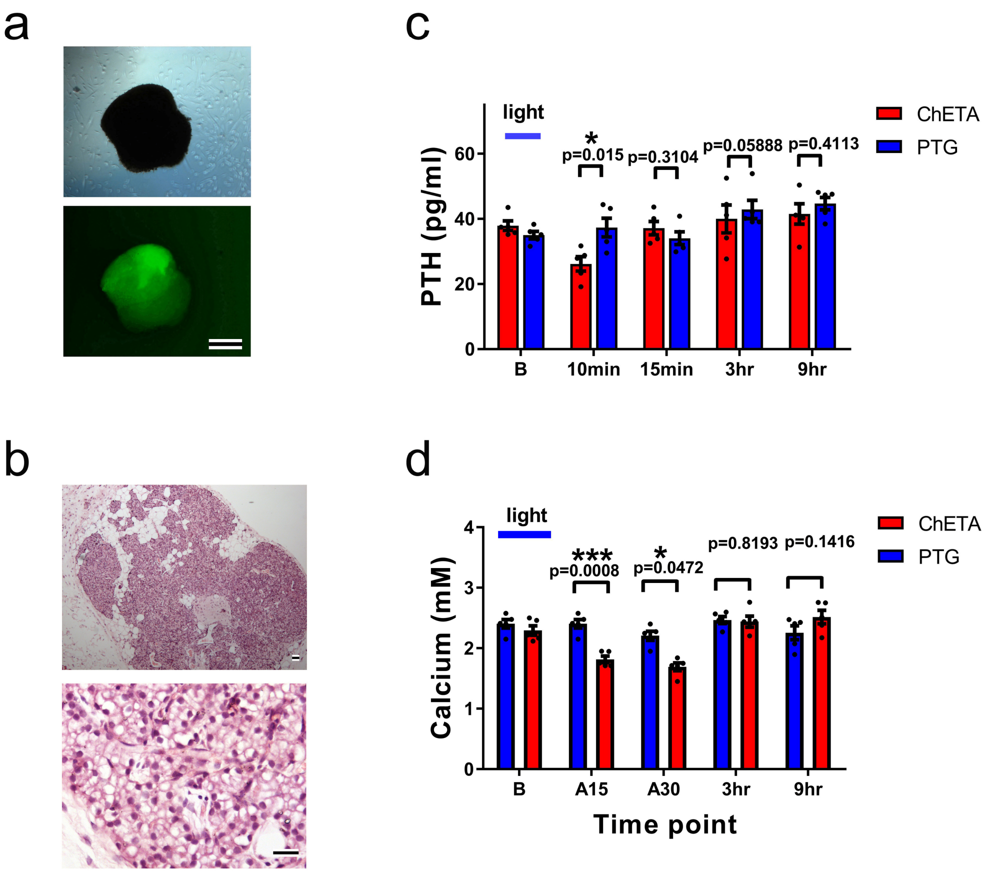

Supplementary Fig. 15 Histology of transplanted parathyroid tissue and optogenetic inhibition of human PTH release in nude mice

a, Green fluorescence was continuously observed in the ChETA-transfected human parathyroid gland tissue before the transplantation. Scale bar=200 μm. **b,** HE staining shows the intact and homogenous cellularity of the human parathyroid tissue following light stimulation. Scale bar=50 μm. **c**, Quantification of PTH levels in the ChETA and PTG groups before (B) and at 10 min, 15 min, 3 h and 9 h after light stimulation (n=5 per group). Two-tailed unpaired *t* test, *p* values as indicated. Values represent mean ±

SEM. **d,** Quantification of calcium levels in the ChETA and PTG groups before (B) and at 15 min, 30 min, 3 h and 9 h after light stimulation. Serum calcium was lower in ChETA group than the control group at 15 and 30 min after blue light stimulation (n=5 per group). Two-tailed unpaired *t* test, *p* values as indicated. Values represent mean ± SEM. Source data are provided as a Source Data file.

Supplementary Figure. 16

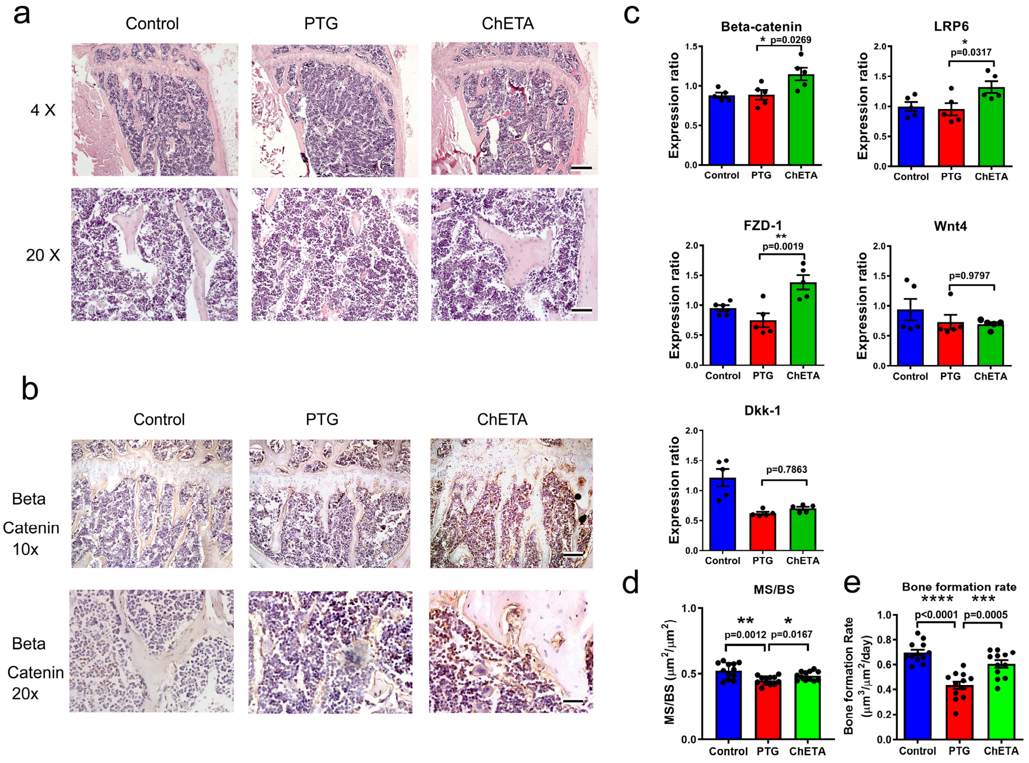

Supplementary Fig. 16 Histology and molecular changes of bone in different experimental groups after optogenetic inhibition of PTH release.

a, HE staining showing trabecular bone structure in the control, PTG (transplanted PTG without transfection) and ChETA (transplanted with ChETA-transfected PTG) groups. Lower bone mass and less trabecular bone structure were observed in the PTG group than in the control group; however, in the ChETA group, bone mass increased notably and the trabecular bone structure recovered. Scale bar=100 μm. **b**, Beta-catenin expression in the control, PTG and ChETA groups after optogenetic inhibition of PTH release. In the PTG group, the signals in trabecular bone were lower than in the control group and positive signals were mainly distributed in the bone marrow cavity. However,

in the ChETA group, intense beta-catenin signals were observed again both in the trabecular bone area and marrow cavity; scale bar=100 μm. **c**, RT-PCR analysis of *beta-catenin*, *LRP6*, *FZD1*, *Wnt4* and *Dkk-1* in the control, PTG and ChETA groups after optogenetic inhibition of PTH release. Values represent mean ±SEM (n=5 mice per group). **d**, The mineralizing surface vs. the bone surface in the control, PTG and ChETA groups. Values represent mean ±SEM (n=12 per group). **e**, Bone formation rate in the control, PTG and ChETA groups. Values represent mean ±SEM (n=12 per group). All statistical tests in **c-e** used: one-way analysis of variance (ANOVA) with Tukey's multiple comparisons test, *p* values as indicated. Values represent mean ± SEM. Source data are provided as a Source Data file.

Supplementary Figure. 17

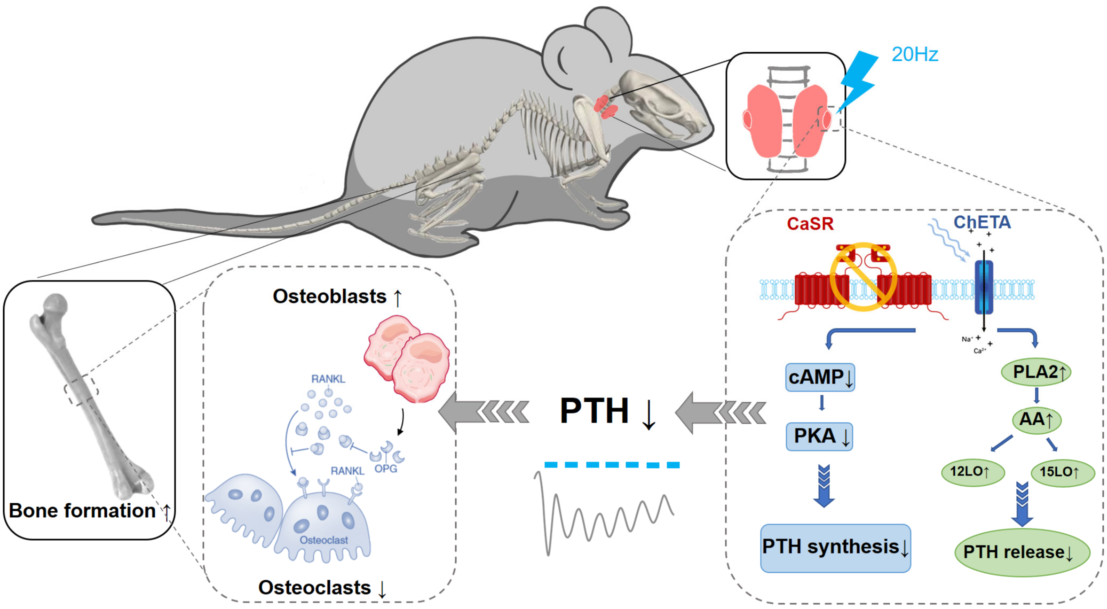

Supplementary Fig. 17 Schematic summarizing the mechanism underlying optogenetic regulation of rhythmic PTH release showing that rhythmic inhibition of PTH was successfully used to enhance bone formation and inhibit bone resorption.
