# Supplementary material for: An optogenetic approach for regulating human parathyroid hormone secretion
Source: Nat Commun. 2022 Feb 9;13:771. doi: 10.1038/s41467-022-28472-9 (PMC8828854; doi:10.1038/s41467-022-28472-9)
Supplement: Supplementary file 3 — Reporting Summary [file 41467_2022_28472_MOESM3_ESM.pdf]

nature research

Corresponding author(s): Fan Yang

Last updated by author(s): Oct 25, 2021

Reporting Summary

Nature Research wishes to improve the reproducibility of the work that we publish. This form provides structure for consistency and transparency in reporting. For further information on Nature Research policies, see Authors & Referees and the Editorial Policy Checklist .

Statistics

For all statistical analyses, confirm that the following items are present in the figure legend, table legend, main text, or Methods section.

n/a	Confirmed	
☐	☒	The exact sample size (*n*) for each experimental group/condition, given as a discrete number and unit of measurement
☐	☒	A statement on whether measurements were taken from distinct samples or whether the same sample was measured repeatedly
☐	☒	The statistical test(s) used AND whether they are one- or two-sided<br>*Only common tests should be described solely by name; describe more complex techniques in the Methods section.*
☐	☒	A description of all covariates tested
☐	☒	A description of any assumptions or corrections, such as tests of normality and adjustment for multiple comparisons
☐	☒	A full description of the statistical parameters including central tendency (e.g. means) or other basic estimates (e.g. regression coefficient) AND variation (e.g. standard deviation) or associated estimates of uncertainty (e.g. confidence intervals)
☐	☒	For null hypothesis testing, the test statistic (e.g. *F*, *t*, *r*) with confidence intervals, effect sizes, degrees of freedom and *P* value noted<br>*Give P values as exact values whenever suitable.*
☒	☐	For Bayesian analysis, information on the choice of priors and Markov chain Monte Carlo settings
☒	☐	For hierarchical and complex designs, identification of the appropriate level for tests and full reporting of outcomes
☒	☐	Estimates of effect sizes (e.g. Cohen's *d*, Pearson's *r*), indicating how they were calculated

Our web collection on statistics for biologists contains articles on many of the points above.

Software and code

Policy information about availability of computer code

Data collection	Skyscan MicroCT software 2.6, pClamp 10, Zen softwares 2.3, Adobe Photoshop CC2015
Data analysis	Skyscan NRecon 1.6.8.0, Skyscan DataViewer 1.4.4.0, Skyscan CTAn 1.13.2.1, Skyscan CTVol 2.2.3.0, ImageJ (V1.51t), Prism 7.0, Statview version 10.0 and Origin 7.5 were used for analysis and statistical assessments

For manuscripts utilizing custom algorithms or software that are central to the research but not yet described in published literature, software must be made available to editors/reviewers. We strongly encourage code deposition in a community repository (e.g. GitHub). See the Nature Research guidelines for submitting code & software for further information.

Data

Policy information about availability of data

All manuscripts must include a data availability statement. This statement should provide the following information, where applicable:

- Accession codes, unique identifiers, or web links for publicly available datasets
- A list of figures that have associated raw data
- A description of any restrictions on data availability

All data generated in this study that support our findings are presented within this paper, its Supplementary Information, or in the source data. All additional information will be made available upon reasonable request to the corresponding author. Source data are provided with this paper.

Field-specific reporting

Please select the one below that is the best fit for your research. If you are not sure, read the appropriate sections before making your selection.

Life sciences study design

All studies must disclose on these points even when the disclosure is negative.

Sample size	No statistical methods were used to pre-determine sample size. The sample sizes of each set of animals were determined according to previous studies performed by our group and other scholars and were fixed in a prospective manner. The exact number of animal used in individual experiments are indicated in the figure legends.
Data exclusions	No data were excluded from the analyses
Replication	All experiments were independently replicated at least three times and the data were reproducible between each study.
Randomization	All samples and animals used in the study were randomly allocated into different experimental groups.
Blinding	The investigators were blinded to group allocation during data collection and analyses.

Reporting for specific materials, systems and methods

We require information from authors about some types of materials, experimental systems and methods used in many studies. Here, indicate whether each material, system or method listed is relevant to your study. If you are not sure if a list item applies to your research, read the appropriate section before selecting a response.

Materials & experimental systems

n/a	Involved in the study	
☐	☒	Antibodies
☒	☐	Eukaryotic cell lines
☒	☐	Palaeontology
☐	☒	Animals and other organisms
☐	☒	Human research participants
☒	☐	Clinical data

Methods

n/a	Involved in the study	
☒	☐	ChIP-seq
☒	☐	Flow cytometry
☒	☐	MRI-based neuroimaging

Antibodies

Antibodies used	Details on the antibodies used in this study are described in the Methods section. Primary antibodies to anti-CaSR (ab18200, Abcam), anti-PTH (ab14493, Abcam), anti-VDR (ab115495, Abcam), anti-Tuj1 (ab18207, Abcam), anti-alpha smooth muscle Actin antibody [1A4] (ab7817, Abcam). anti-ALP (ab95462, abcam), anti-Beta-catenin (C2206, Sigma). The sections were then washed and labeled with fluorescence-conjugated corresponding secondary antibodies Alexa Fluor@ 488-conjugated goat anti-rabbit (111-547-003, Jackson ImmunoResearch), Alexa Fluor@ 488-conjugated goat anti-mouse (115-547-003, Jackson ImmunoResearch), Alexa Fluor@594-conjugated goat anti-rabbit (111-587-003, Jackson ImmunoResearch) or Alexa Fluor@594-conjugated goat anti-mouse IgG antibodies (115-587-003, Jackson ImmunoResearch) as the secondary antibodies. For dilution fold, primary antibodies were used at 1:500 (unless otherwise stated elsewhere), with 2nd antibodies used at 1:100 (unless otherwise stated elsewhere).
Validation	All the antibodies were validated for use in mouse/rat/human tissues based on previous publications or preliminary tests; anti-CaSR (ab18200) by Zhang X et al. Front Physiol (2018); anti-PTH (ab14493), RRID: AB_301271, was validated by our preliminary tests. anti-VDR (ab115495), RRID:AB_10903196, was validated by our preliminary tests; anti-Tuj1 (ab18207) by Dolan CP et al. Dev Biol (2019); anti-alpha smooth muscle actin (ab7817) by Zheng Q et al. J Cell Mol Med (2019); anti-ALP (ab95462) by Li Z et al. Cell Death Dis (2019); anti-beta-catenin (C2206) by Liang CJ et al. Cell Rep (2019); Alexa Fluor 594-AffiniPure Goat Anti-Rabbit (111-585-003) by Wang M et al. Cell Stem Cell (2018); Alexa Fluor 488-AffiniPure Fab Fragment Goat Anti-Rabbit (111-547-003) by Joseph DB et al. Dev Biol (2019); Alexa Fluor 594-AffiniPure Goat Anti-Mouse (115-585-003) by Wang M et al. Cell Stem Cell (2018); Alexa Fluor 488-AffiniPure Fab Fragment Goat Anti-Mouse (115-547-003) by Joseph DB et al. Dev Biol. (2019); More details of antibodies' validation profiles are available in the website of designated companies

Animals and other organisms

Policy information about studies involving animals; ARRIVE guidelines recommended for reporting animal research

Laboratory animals	Adult male C57BL/6 (6 to 8 week-old), male Sprague Dawley rat (8-10 week-old) and male CD-1 nude mice (8-10 week-old) were purchased from Guangdong Medical Laboratory Animal Center, Guangzhou, China. Mice and rat were housed at 22-25 °C, 40–60% humidity on a circadian cycle of 12-hour light and 12-hour dark with ad-libitum access to food and water.
Wild animals	No wild animal were used in this study
Field-collected samples	No field-collected samples were used in this study

Ethics oversight	Animal care and experimental procedures were performed with approval by the Research Committee of the Shenzhen Institutes of Advanced Technology, Chinese Academy of Sciences. The protocol number is SIAT-IRB-160909-NS-YANGF-A0237.

Note that full information on the approval of the study protocol must also be provided in the manuscript.

Human research participants

Policy information about studies involving human research participants

Population characteristics	In total, 10 parathyroid glands were collected from 8 patients who had diagnosed with secondary hyperparathyroidism and undergone parathyroidectomy surgery at Shenzhen People's Hospital (Shenzhen, China). The detailed clinicopathological characteristics were described in Tables 1. All tissue specimens were collected from August 2017 to February 2019 with the consent of patients and approved by Ethics Committee of Shenzhen People's Hospital.
Recruitment	Participants were recuited from the pool of patients diagnosed with secondary hyperparathyroidism at Shenzhen People's Hospital. No selection bias was observed.
Ethics oversight	All tissue specimens were collected from August 2017 to February 2019 with the consent of patients and approved by Ethics Committee of Shenzhen People's Hospital. Patient informed consent form was properly signed before recruitment for the study.

Note that full information on the approval of the study protocol must also be provided in the manuscript.
